# Whipple's Disease: A Textbook Disease That Is Often Missed in Real Life

**DOI:** 10.14309/crj.0000000000001199

**Published:** 2023-11-03

**Authors:** Yuting Huang, Stevi M. Harrison, Aman Bali, Ruchit Rangani, Hamza Ashmila, Dilhana S. Badurdeen

**Affiliations:** 1 Division of Gastroenterology & Hepatology, Mayo Clinic in Florida, FL; 2 Department of Medicine, Mayo Clinic in Florida, FL; 3 Department of Pathology, Mayo Clinic in Florida, FL

## CASE REPORT

**Figure 1:**
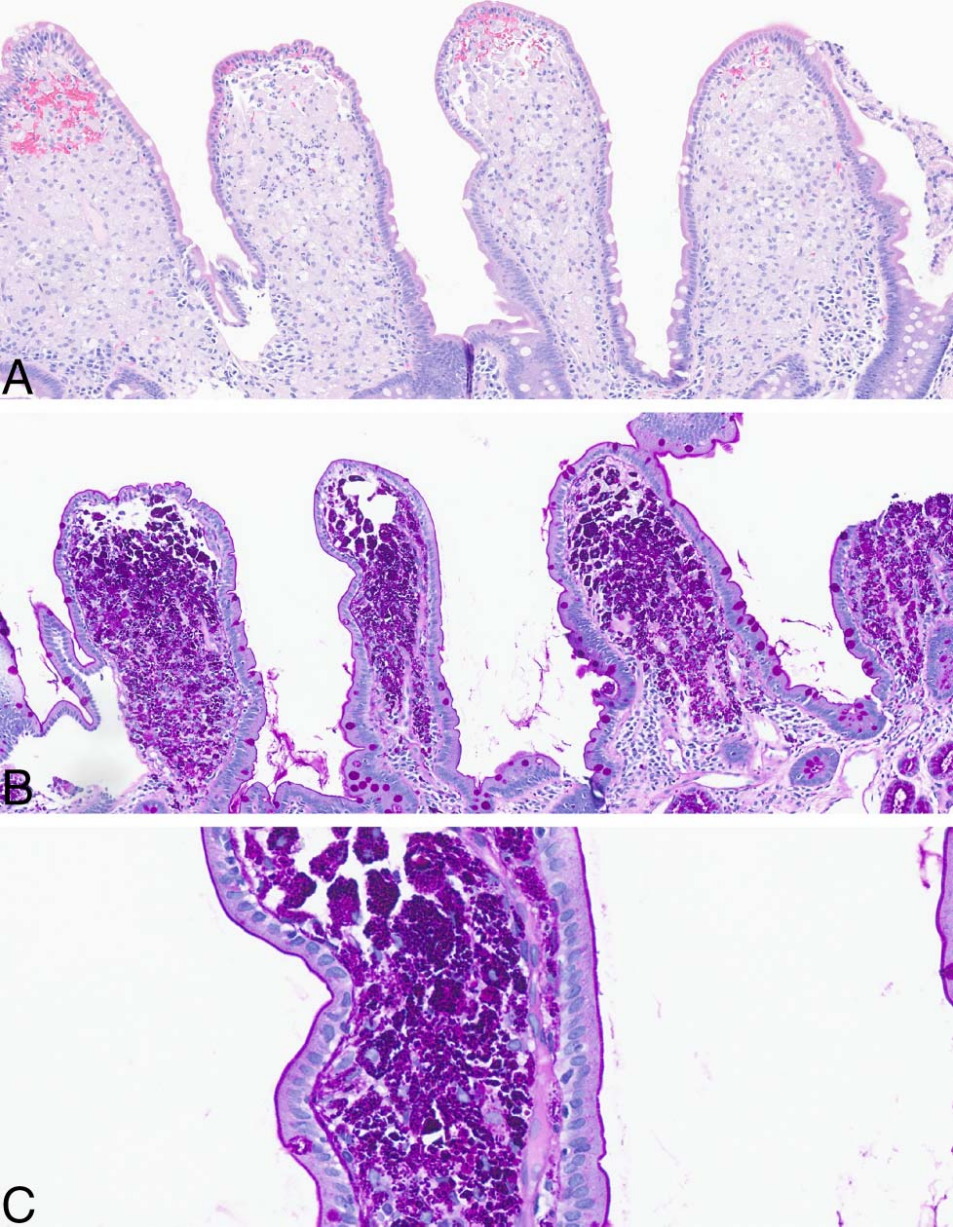
Informed consent was obtained for this case report. Pathology examination of duodenal and terminal ileal biopsies showed partial villous flattening and intraepithelial lymphocytosis (A, hematoxylin and eosin ×20). Periodic acid-Schiff stain highlighted intracellular organisms in macrophages within the lamina propria (B, ×20; C, ×60).

A 54-year-old man who worked as a farmer with history of sarcoidosis not on treatment presented with 1 year of diarrhea (20+ watery bowel movements daily) and unintentional weight loss of ∼100 lbs without extragastrointestinal symptoms. Workup for gastrinoma, *Strongyloides*, and celiac disease was negative. The patient had normal abdominal computed tomography with intravenous contrast. Stool pathogen polymerase chain reaction was positive for *Giardia* and non-choleric *Vibrio* species; reflex stool *Giardia* antigen and stool culture for *Vibrio* were negative. The patient was treated empirically with nitazoxanide and pancrealipase without clinical improvement. Whipple disease was diagnosed with positive serum *Tropheryma whipplei* polymerase chain reaction. Esophagogastroduodenoscopy showed gastritis. Pathology showed partial villous flattening, moderate intraepithelial lymphocytosis, and periodic acid-Schiff-positive intracellular organisms in macrophages in the duodenum and terminal ileum (Figure 1). The patient was given intravenous ceftriaxone for 4 weeks, followed by oral trimethoprim-sulfamethoxazole for 1 year. His diarrhea resolved, and he gained 60 lbs over the next 12 months.

Whipple disease is extremely rare, mostly seen in middle-aged White men in North America and Europe who work closely with soil, sewage, and wastewater. It frequently coexists with other gastrointestinal infections so is misdiagnosed. Delayed diagnosis and treatment results in fatal outcomes, including central nervous system infection. It is crucial to test for and treat Whipple disease in high-risk populations.

## DISCLOSURES

Author contributions: Y. Huang: case presentation, figure visualization, manuscript writing and editing. SM Harrison: case presentation, manuscript drafting. A. Bali: case presentation, manuscript drafting. R. Rangani: case presentation. H. Ashmila: pathology interpretation. DS Badurdeen: supervising, manuscript finalization. DS Badurdeen is the article guarantor.

Financial disclosure: None to report.

